# Effects of Repetitive Compressive Loading on the Expression of Autophagy-Related Factors, Autophagy Capacity and Cellular Activity in Human Subchondral Bone Osteoblasts

**DOI:** 10.3390/ijms27219903

**Published:** 2026-11-05

**Authors:** Hideaki Iwata, Satomi Sato, Shu Somemura, Masahiro Takemoto, Yuki Takahashi-Suzuki, Yodo Sugishita, Hiroto Fujiya, Naoki Haraguchi, Kazuo Yudoh

**Affiliations:** 1 Graduate School, St. Marianna University School of Medicine, Sugao 2-16-1, Miyamae-ku, Kawasaki 216-8512, Japan; hideaki.iwata@marianna-u.ac.jp (H.I.); satomi.kimura@marianna-u.ac.jp (S.S.); 2 Department of Orthopedic Surgery, St. Marianna University School of Medicine, Sugao 2-16-1, Miyamae-ku, Kawasaki 216-8512, Japan; s3somemura@marianna-u.ac.jp (S.S.); masahiro.takemoto@marianna-u.ac.jp (M.T.); naoki.haraguchi@marianna-u.ac.jp (N.H.); 3 Department of Sports Medicine, St. Marianna University School of Medicine, Sugao 2-16-1, Miyamae-ku, Kawasaki 216-8511, Japan; fujiya-1487@marianna-u.ac.jp; 4 Department of Frontier Medicine, Institute of Medical Science, St. Marianna University School of Medicine, Sugao 2-16-1, Miyamae-ku, Kawasaki 216-8512, Japan; y2takahashi@marianna-u.ac.jp (Y.T.-S.); yodo@marianna-u.ac.jp (Y.S.)

**Keywords:** osteoblasts, subchondral bone, mechanical stress, autophagy, mitochondria

## Abstract

Bone metabolism is known to change in response to mechanical stress. In osteoarthritis (OA), the subchondral bone as well as the articular cartilage layer is damaged by mechanical stress, which adversely affects the mechanical environment and homeostatic balance of the entire joint, leading to the onset of OA. However, the impact of mechanical stress on osteoblasts in the subchondral bone, especially their defense mechanism against stress, remains unclear. We focused on the mechanical stress response capacity of subchondral bone osteoblasts, which, like chondrocytes in the cartilage layer, are routinely exposed to repetitive mechanical stress. The purpose of this study was to determine whether repetitive mechanical force (at a physiologic level of cyclic compressive loading) affects the expression of factors regulating autophagy, autophagy capacity and cellular activity in subchondral osteoblasts. Three-dimensional cultured tissue was generated from human subchondral bone osteoblasts using a collagen sponge scaffold. Repetitive compressive loading was applied to the 3D cell culture constructs. After mechanical loading on 3D cell–collagen sponge constructs, we comparatively analyzed the expression of autophagy-related factors (ATG5, Beclin-1, Parkin) in human osteoblasts. Levels of osteoblast differentiation activity (alkaline phosphatase) were also analyzed, and transmission electron microscopy (TEM) was used to detect autophagy processes with or without the repetitive compressive loading. In both premature and mature osteoblasts, repetitive compressive loading decreased expression of the autophagy-related factors ATG5, Beclin-1 and Parkin in comparison with the non-loading group, suggesting that repetitive compressive loading may induce downregulation of mitophagy as well as cellular autophagy in subchondral osteoblasts. TEM analysis indicated that, in both premature and mature osteoblasts of the non-loading group, autophagy processes were progressing. In contrast, repetitive loading decreased the number of autophagosomes and autolysosomes in mature and premature osteoblasts. Our results demonstrated that repetitive mechanical loading resulted in a reduction in cellular autophagy and mitophagy in both premature and mature osteoblasts, while also inducing osteoblast differentiation. While mechanical stress promotes osteoblast differentiation, it also alters autophagy, a cellular defense mechanism, suggesting that under conditions of sustained repetitive loading, alterations in the autophagy response—which is essential for maintaining intracellular homeostasis—occur alongside the adaptive bone-forming response, potentially affecting osteoblast function in the long term. Our data demonstrate that reductions in both mitophagy and cellular autophagy in response to repetitive mechanical stress, even at a physiologic level, may lead to accumulation of defective mitochondria and abnormal cellular proteins in the cells, thus reducing osteoblast activity and affecting the homeostasis of subchondral bone tissue, and ultimately contributing to OA progression.

## 1. Introduction

In modern aging societies, the incidence of age-associated osteochondral conditions—such as osteoporosis, fragility fractures, and osteoarthritis (OA)—is escalating rapidly, contributing significantly to patient morbidity and long-term bedridden rates [1,2]. Globally, osteoporosis affects an estimated 18.3% of the population, with secondary fragility fractures reducing post-fracture survival rates by approximately 20% [3,4,5]. In Japan alone, an estimated 15 million individuals (nearly 10% of the total population) suffer from osteoporosis, with approximately 193,400 hip fractures occurring annually [6,7]. Concurrently, OA represents a major degenerative joint disorder characterized by progressive cartilage degradation, secondary synovial inflammation, and subchondral bone remodeling, leading to chronic pain and loss of physical mobility. Epidemiological surveys in Japan indicate that over 25 million people have radiographically diagnosed OA, with more than 12.8 million presenting with clinical symptoms, and approximately 900,000 new diagnoses recorded each year [8].

In recent years, the pathological contribution of subchondral bone degeneration to OA progression has gained widespread recognition. Similar to its damaging effects on articular cartilage, chronic repetitive mechanical strain impacts subchondral osteoblasts, serving as a primary driver of osteochondral degeneration [9,10]. Developing effective preventive and therapeutic interventions for metabolic bone disorders, fractures, and OA requires a clear understanding of the molecular mechanisms governing bone metabolism. Bone turnover continuously adapts to diverse intrinsic and extrinsic environmental stressors, including inflammatory signals, mechanical forces, cellular senescence, genomic instability, hormonal shifts, and nutritional status [9,10,11]. Among these factors, mechanical loading stands out as a fundamental regulator of bone tissue metabolism [10,11].

When physical strain is exerted on bone tissue, biophysical electrical potential changes are generated, directing the spatial activity of osteoblasts and osteoclasts, coupling bone synthesis with resorption, and accelerating overall bone metabolic rate. Consequently, various physical modalities, including electromagnetic field exposure, have been extensively investigated for their capacity to modulate bone turnover [12,13]. Clinical application has validated these biophysical stimuli, leading to official regulatory approval of electromagnetic field and ultrasound therapies for accelerating fracture healing and non-union repair [12]. Research continues to unravel the specific mechanotransduction networks that mediate these metabolic adaptations.

At the cellular level, the molecular cascades underlying mechanical stress-induced bone formation are increasingly understood [9]. Osteocytes entrained within the lacunocanalicular network interact directly with osteoblasts on the bone surface through extended dendritic processes, regulating bone turnover via intercellular communication [9,10,11,14,15]. Osteocytes primarily modulate osteoblastic bone-forming capacity through the canonical Wnt signaling pathway [16,17,18,19,20,21]. Upon sensing mechanical strain, osteocytes downregulate their expression and secretion of sclerostin, an endogenous Wnt antagonist [22,23]. The resulting relief of Wnt inhibition unleashes osteoblastic Wnt signaling, thereby promoting osteoblast differentiation, activity, and matrix mineralization [22,23]. These observations highlight the critical role of osteocyte–osteoblast intercellular signaling in transducing mechanical forces into bone structural adaptation.

In addition to osteocyte-mediated regulation, osteoblasts possess cell-autonomous mechanosensing mechanisms that directly enhance their functional capacity. We previously demonstrated that mechanical strain applied to osteoblasts triggers cell-membrane glucose transporter 1 (Glut1) activation, driving intracellular glucose uptake [24]. This metabolic adaptation suppresses sirtuin 1 (SIRT1), a key mitochondrial deacetylase, which subsequently derepresses the osteogenic master transcription factor Runx2—a factor negatively regulated by SIRT1 [24]. Activation of this Glut1–SIRT1–Runx2 pathway accelerates osteoblast differentiation, maturation, and matrix synthetic capacity, confirming that mechanical forces directly reconfigure energy metabolism and mitochondrial regulatory axes in osteoblasts [24]. Nevertheless, how osteoblasts directly perceive mechanical strain and maintain cellular integrity in the absence of osteocytic dendritic networks remains incompletely understood, particularly regarding intrinsic protective defense mechanisms in subchondral bone.

To survive environmental stressors, cells rely on macroautophagy (hereafter termed autophagy) as a critical homeostatic defense mechanism [25,26,27]. By sequestering and recycling essential cytoplasmic components, as well as clearing toxic protein aggregates and damaged organelles, autophagy maintains cellular metabolic balance; conversely, autophagic dysfunction leads to mitochondrial decay, cellular senescence, and diverse age-related pathologies [28,29]. During autophagy, cellular waste and dysfunctional mitochondria emit specific stress signals that initiate the nucleation of double-membrane autophagosomes around targeted cargo [29]. These autophagosomes subsequently fuse with hydrolase-rich lysosomes, resulting in enzymatic degradation of the enclosed material and the release of basic biomolecules, such as amino acids, to sustain energetic demand and organelle renewal [29].

The organelle quality control function of autophagy has been extensively investigated in articular chondrocytes during OA pathogenesis. Chondrocytes utilize autophagy and selective mitochondrial degradation (mitophagy) as vital defense systems against stress-induced cellular damage and mitochondrial failure, ensuring the elimination of non-functional, ATP-deficient mitochondria [25,26,30,31]. This selective breakdown safeguards mitochondrial quality and cellular homeostasis in articular cartilage [32,33]. Impairment of autophagic flux is well documented in osteoarthritic chondrocytes compared with healthy controls, directly implicating autophagic failure in matrix degradation and joint failure [32,34,35]. Notably, our recent investigation established that repetitive mechanical loading suppresses key autophagic regulatory factors in human chondrocytes, compromising cellular activity and contributing to cartilage breakdown [36].

Concurrently, subchondral bone degeneration is increasingly recognized as an active contributor to OA development [37,38,39]. The subchondral plate and trabecular bone form a functionally coupled osteochondral unit with the overlying articular cartilage, jointly maintaining biomechanical shock absorption and joint homeostasis [39,40]. Pathological or repetitive mechanical loading disrupts this functional unit, accelerating osteochondral degradation and OA clinical onset [37,39,40]. Subchondral bone cells, including osteoblasts and osteocytes, likely depend on autophagic protection to counter stress-induced organelle damage and metabolic decline. It is plausible that subchondral osteoblasts undergo mechanical stress-induced autophagic alterations similar to those we identified in articular chondrocytes [36]. However, whether repetitive mechanical stress directly impairs autophagic capacity in subchondral osteoblasts, leading to dysfunctional mitochondrial accumulation and subchondral bone metabolic dysfunction, remains uncharacterized.

The core molecular pathway of autophagy involves highly conserved regulatory proteins [28,41,42,43,44]. Autophagy-related protein 5 (ATG5) is indispensable for autophagosome membrane elongation and also participates in cytoskeletal reorganization and apoptotic signaling [44,45]. Beclin-1 functions as an essential initiator of autophagy induction by forming a complex with Vps34 and Vps15 to activate class III phosphatidylinositol 3-kinase activity [46,47]. Distinct from ATG5 and Beclin-1, the E3 ubiquitin ligase Parkin specifically governs mitophagy [48,49,50]. Upon mitochondrial depolarization, Parkin translocates to the outer mitochondrial membrane, ubiquitinating targeted outer-membrane proteins to recruit proteasomal and autophagic machinery for defective organelle clearance [48,49,50].

We hypothesized that in the subchondral bone layer, repetitive mechanical loading downregulates cellular autophagy and Parkin-mediated mitophagy in osteoblasts, leading to the intracellular accumulation of damaged mitochondria and defective protein aggregates, which impairs osteoblast functional activity and subchondral bone health, ultimately driving osteochondral degeneration in OA. To test this hypothesis, we utilized a 3D osteoblast-collagen culture model subjected to cyclic compressive loading to evaluate how repetitive mechanical stress impacts autophagic capacity and the expression of ATG5, Beclin-1, and Parkin in human subchondral osteoblasts.

## 2. Results

### 2.1. Operational Parameters and System Characterization for Dynamic Compression

**Figure 1:**
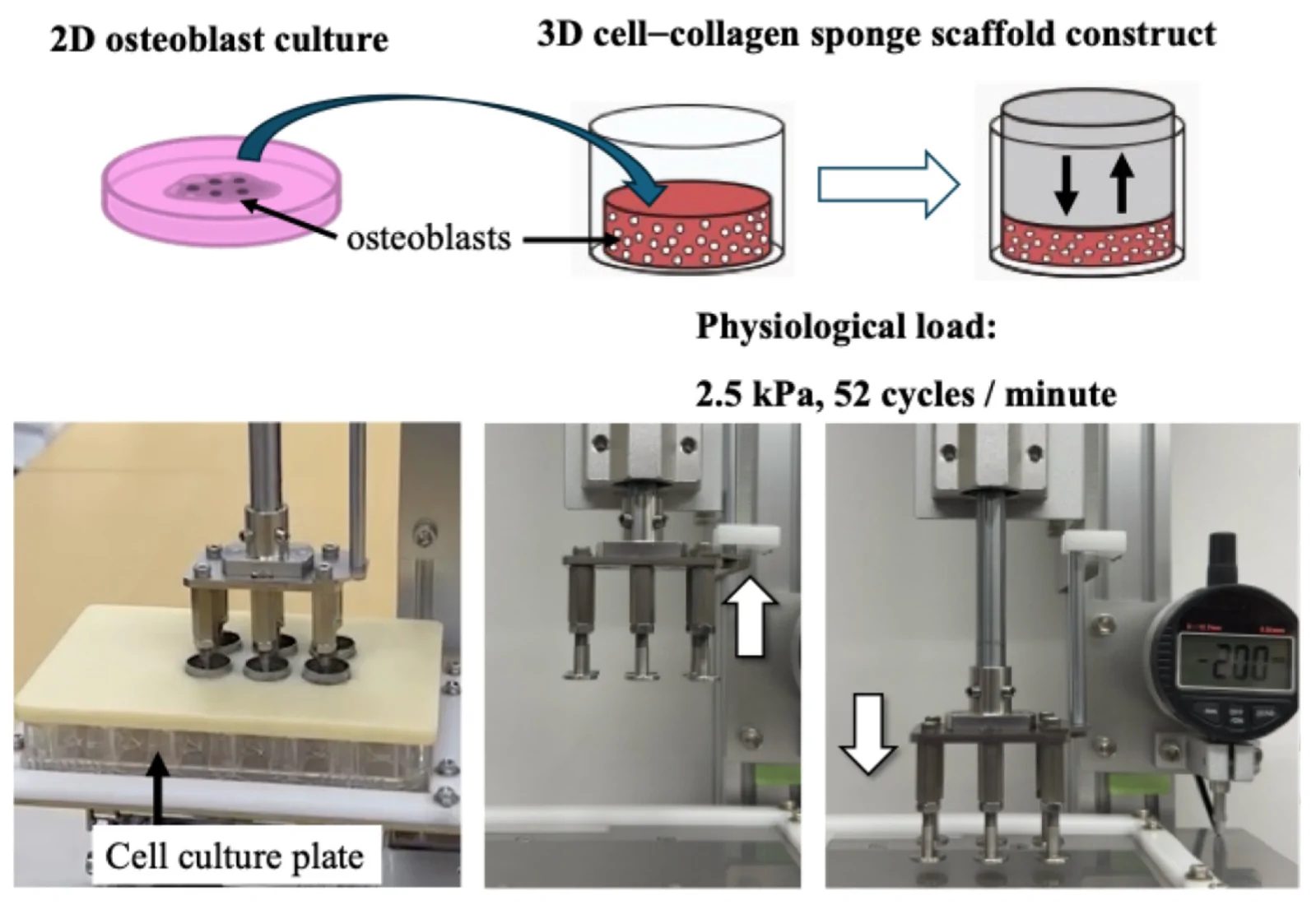
Preparation and mechanical loading setup of 3D human osteoblast–collagen scaffold constructs.

Three-dimensional cell–scaffold constructs were positioned within individual wells of 12-well culture plates containing 1.5 mL of Dulbecco’s modified Eagle medium. Mechanical strain was applied to the 3D constructs using a custom-engineered compression device (Figure 1).

Physiological cyclic loading was administered at either 0 kPa (unloaded baseline) or 2.5 kPa (25.5 gf/cm^2^). In line with our preliminary trials and prior reports, compressive stress at this physiological intensity caused no detectable cellular injury or apoptotic cell death, confirming the suitability of 2.5 kPa (25.5 gf/cm^2^) for all subsequent experimental loading conditions [24,36,51].

Preparatory evaluations conducted during system characterization established the following operational features:

(i) The device delivered a sinusoidal compressive wave with a peak magnitude of 2.5 kPa at 0.87 Hz, generating internal compressive strains ranging from several tenths of a percent up to approximately 1%.
(ii) Each compression cycle spanned 1.15 s, closely matching the rhythm of single-leg loading during slow locomotion or cardiac-driven movement. Accordingly, a frequency of 52 cycles/minute (equivalent to 0.87 Hz) was adopted.
(iii) Compressive forces within this physiological threshold have been validated by existing literature as non-cytotoxic to osteoarticular tissue constructs [24,36,51,52].
(iv) Mechanical performance metrics—including torque output, cycle-to-cycle consistency, and stroke velocity—were monitored continuously across extended time points (15 min, 30 min, 1 h, 6 h, 12 h, 24 h, and 72 h). System variations remained strictly below 0.5% throughout testing.

Regarding parameter selection, the loading condition was optimized to simulate ambulatory stress rhythms without compromising cell survival or cellular activity within the 3D collagen matrix. Experimental testing confirmed that compressive forces between 2.5 and 4.0 kPa produced no detrimental effects on cell viability. This safety margin aligns with previous literature and is substantiated by our Cell Counting Kit-8 (CCK-8) viability data (Supplementary Data). Consistent with our previous study, no significant difference in CCK-8 cell viability was observed between statically cultured controls and loaded samples [36].

Monolayer-expanded subchondral osteoblasts were harvested and suspended in DMEM at 1.0 × 10^6^ cells/mL. To fabricate 3D tissue constructs, cell suspensions were inoculated onto porous collagen sponges in 96-well culture plates at 1 × 10^5^ cells/scaffold/well1 × 10^5^ cells/scaffold/well and incubated at 37 °C in a humidified atmosphere (95% air/5% CO_2_). Following overnight stabilization, individual 3D cell–collagen discs were transferred into 12-well plates. Dynamic compressive loading was applied at 0 kPa (unloaded control) or 2.5 kPa (25.5 gf/cm^2^) at a frequency of 52 cycles/minute for duration periods of 60 or 180 min. (Representative schematics illustrate scaffold fabrication and the mechanical loading workflow).

### 2.2. Quantitative Evaluation of Cellular Protein Yield Post-Compressive Loading

**Figure 2:**
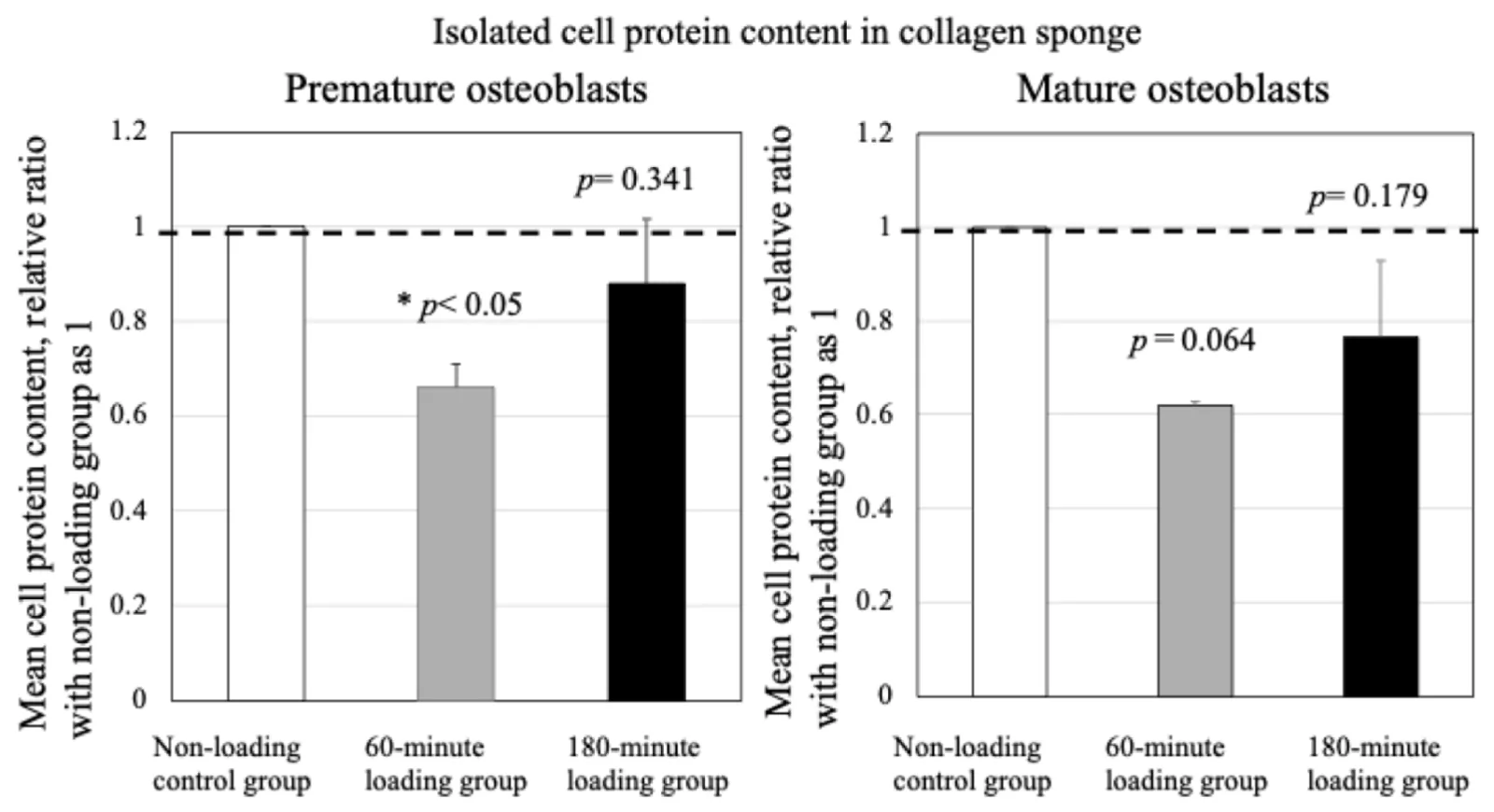
Effects of physiologic and repetitive compressive loading on cell protein content in the 3D osteoblast–collagen construct after mechanical loading. * *p* < 0.05.

As depicted in Figure 2, dynamic loading for 60 min produced a transient decreasing trend in extracted cell protein content from the 3D constructs in both premature osteoblasts (* *p* < 0.05) and mature osteoblasts (*p* = 0.064).

Conversely, extending the duration of repetitive loading to 180 min resulted in no significant reduction in cell protein yield in either premature or mature osteoblasts compared with unloaded controls (premature osteoblasts: *p* = 0.341; mature osteoblasts: *p* = 0.179; Figure 2).

In both premature and mature osteoblasts, application of repetitive and physiologic mechanical stress for 60 min resulted in a decrease in cell protein content between the non-loading control group and the repetitive loading groups (premature osteoblasts: * *p* < 0.05, mature osteoblasts: *p* = 0.064). The 180-min loading showed no significant decrease in cell protein content in the 3D cell-culture construct in comparison with either control in premature or mature osteoblasts. Data represent the mean ± standard deviation from three independent cell-collagen constructs per group.

### 2.3. Induction of Osteogenic Marker Alkaline Phosphatase (ALP) Expression by Mechanical Strain

**Figure 3:**
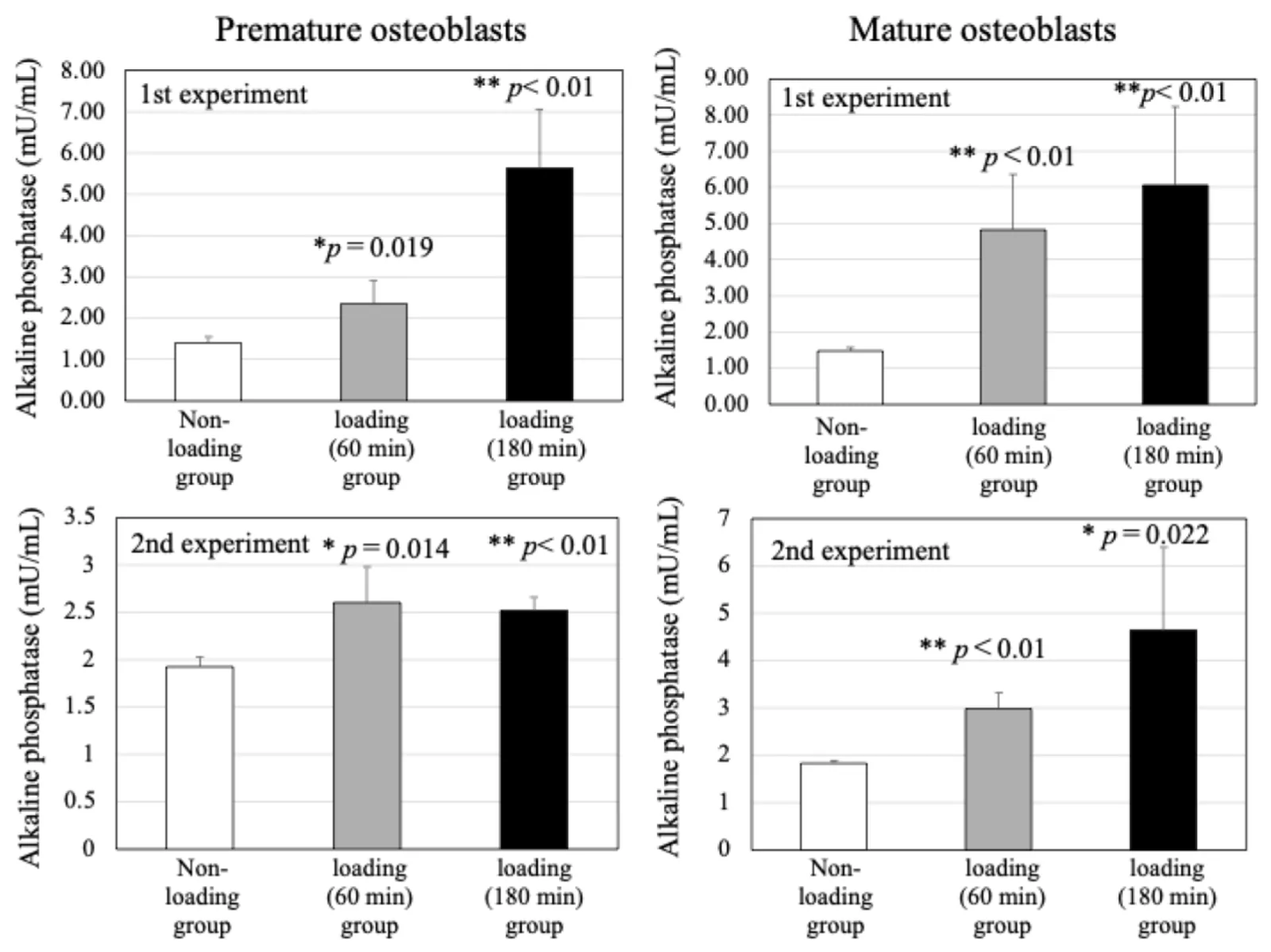
Impact of cyclic compression on alkaline phosphatase (ALP) production in human osteoblasts. Mean ALP levels were significantly elevated in both premature and mature osteoblasts subjected to 60 or 180 min of cyclic loading compared with non-loaded controls (* *p* < 0.05, ** *p* < 0.01). Bars represent the mean ± standard deviation derived from three independent biological constructs per condition.

As shown in Figure 3, subjecting 3D constructs to cyclic compression for either 60 or 180 min significantly elevated the expression of alkaline phosphatase (ALP), a key functional marker of osteoblastic differentiation, in both premature and mature osteoblasts compared with non-loaded controls (*n* = 4 replicates per experiment across four independent biological runs; control vs. 60-min load: *p* < 0.01; control vs. 180-min load: *p* < 0.01; Figure 3). In both differentiation stages, load-induced ALP upregulation progressed in a time-dependent manner, indicating that cyclic mechanical stress promotes osteoblastic functional activity and maturation.

### 2.4. Ultrastructural Assessment of Autophagic Machinery via Transmission Electron Microscopy (TEM)

**Figure 4:**
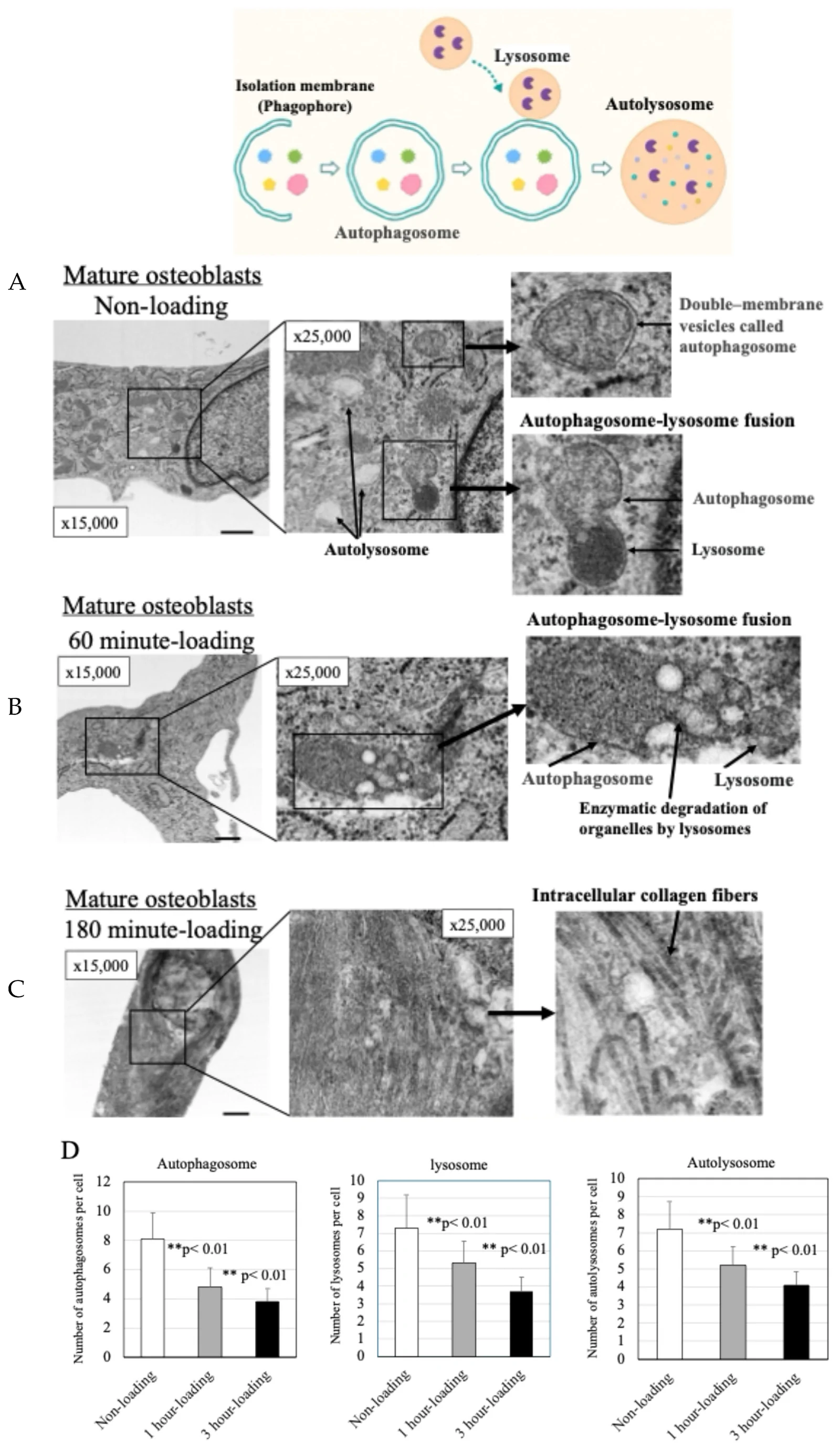

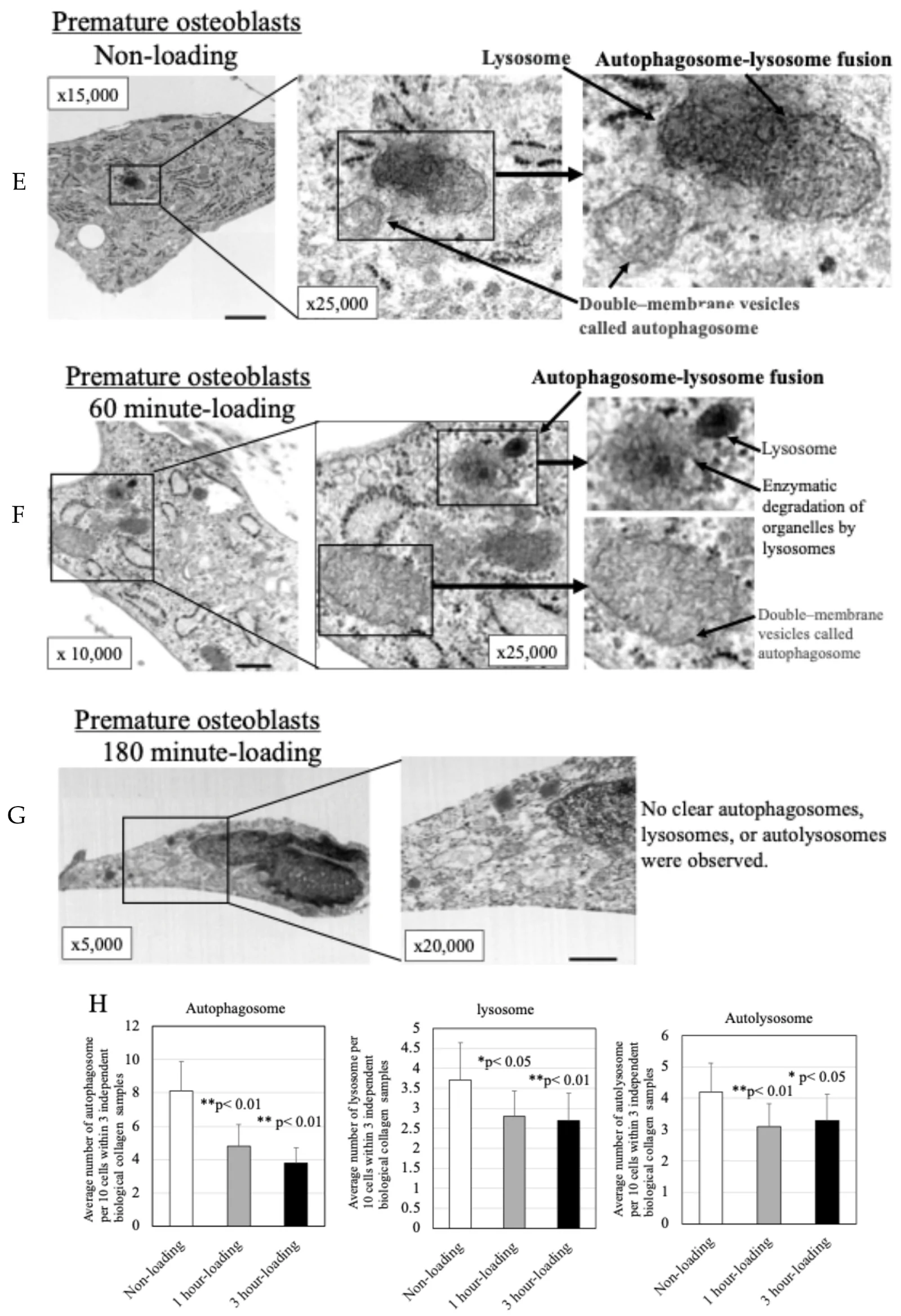
Transmission electron microscopy (TEM) analysis (mature osteoblasts). (**A**): In mature osteoblasts of the non-loading group, TEM revealed many autophagosomes and lysosomes showing the autophagy process, as well as autolysosomes after digestion of organelles. (**B**): In mature osteoblasts of the 60-min loading group, autophagosomes, lysosomes, and autolysosomes were again observed, although fewer in number than in the no-loading group, indicating the autophagy process. (**C**): In mature osteoblasts of the 180-min loading group, very few autophagosomes or autolysosomes were observed, but abundant collagen fiber structures were observed within the cells, suggesting that 180-min loading promoted bone collagen production in mature osteoblasts. (**D**): Quantification of autophagosomes, lysosomes, and autolysosomes in 3D collagen constructs. The graph shows the number of autophagy-related structures per cell. Data represent the mean values per construct (*n* = 3 independent constructs per group; 3–4 cells analyzed per construct, totaling 10 cells per group). Error bars indicate standard deviation. ** *p* < 0.01 (comparison with the unloaded control group using a two-tailed Student’s *t*-test). Evaluation of the number of autophagosomes, lysosomes, and autolysosomes per cell in mature osteoblasts revealed that both the mechanical loading groups showed significantly lower numbers than the non-loading group (** *p* < 0.01). (**E**): TEM analysis of premature osteoblasts of the non-loading group revealed several autophagosomes and lysosomes showing autophagy in progress, as well as autolysosomes after digestion of organelles. (**F**): In premature osteoblasts of the 60-min loading group, the numbers of autophagosomes, lysosomes, and autolysosomes were lower than in premature osteoblasts of the non-loading group. (**G**): In premature osteoblasts of the 180-min loading group, no clear autophagosomes, lysosomes, or autolysosomes were observed. (**H**): Quantification of autophagosomes, lysosomes, and autolysosomes in 3D collagen constructs. The graph shows the number of autophagy-related structures per cell. Data represent the mean values per construct (*n* = 3 independent constructs per group; 3–4 cells analyzed per construct, totaling 10 cells per group). Error bars indicate standard deviation. * *p* < 0.05, ** *p* < 0.01 (comparison with the unloaded control group using a two-tailed Student’s *t*-test). Evaluation of the number of autophagosomes, lysosomes, and autolysosomes per cell in premature osteoblasts showed that there were significantly fewer of these organelles in the mechanical loading groups compared to the non-loading group (* *p* < 0.05, ** *p* < 0.01).

Representative TEM micrographs in Figure 4A,E reveal that under static control conditions, both premature and mature osteoblasts displayed numerous baseline autophagic structures, including autophagosomes, primary lysosomes, autophagosome–lysosome fusion vesicles, and autolysosomes containing partially digested organelles.

In constructs subjected to 60 min of compressive strain, autophagic organelles (autophagosomes, lysosomes, autophagosome–lysosome fusions, and autolysosomes) remained observable in both maturation stages, though their overall frequency was reduced compared with unloaded baselines (Figure 4B,F).

In contrast to the no-loading and 60-min loading groups, very few autophagosomes or autolysosomes were observed in either premature or mature osteoblasts of the 180-min loading group (Figure 4C,G). In the mature osteoblast group, but not in the premature osteoblast group, abundant collagen fiber structures were observed within the cells, suggesting that the 180-min load promoted bone collagen production in mature osteoblasts. In premature osteoblasts of the 180-min loading group, very few autophagosomes, lysosomes, or autolysosomes were observed.

Relative to the control group, these organelle (autophagosomes, lysosomes, and autolysosomes) counts per cell dropped significantly in the 60-min and 180-min loading cohorts (Figure 4D,H, * *p* < 0.05, ** *p* < 0.01). In contrast, no significant variations were observed in organelle size among the subgroups.

### 2.5. Modulation of ATG5 Autophagic Protein Expression Under Cyclic Compressive Force

**Figure 5:**
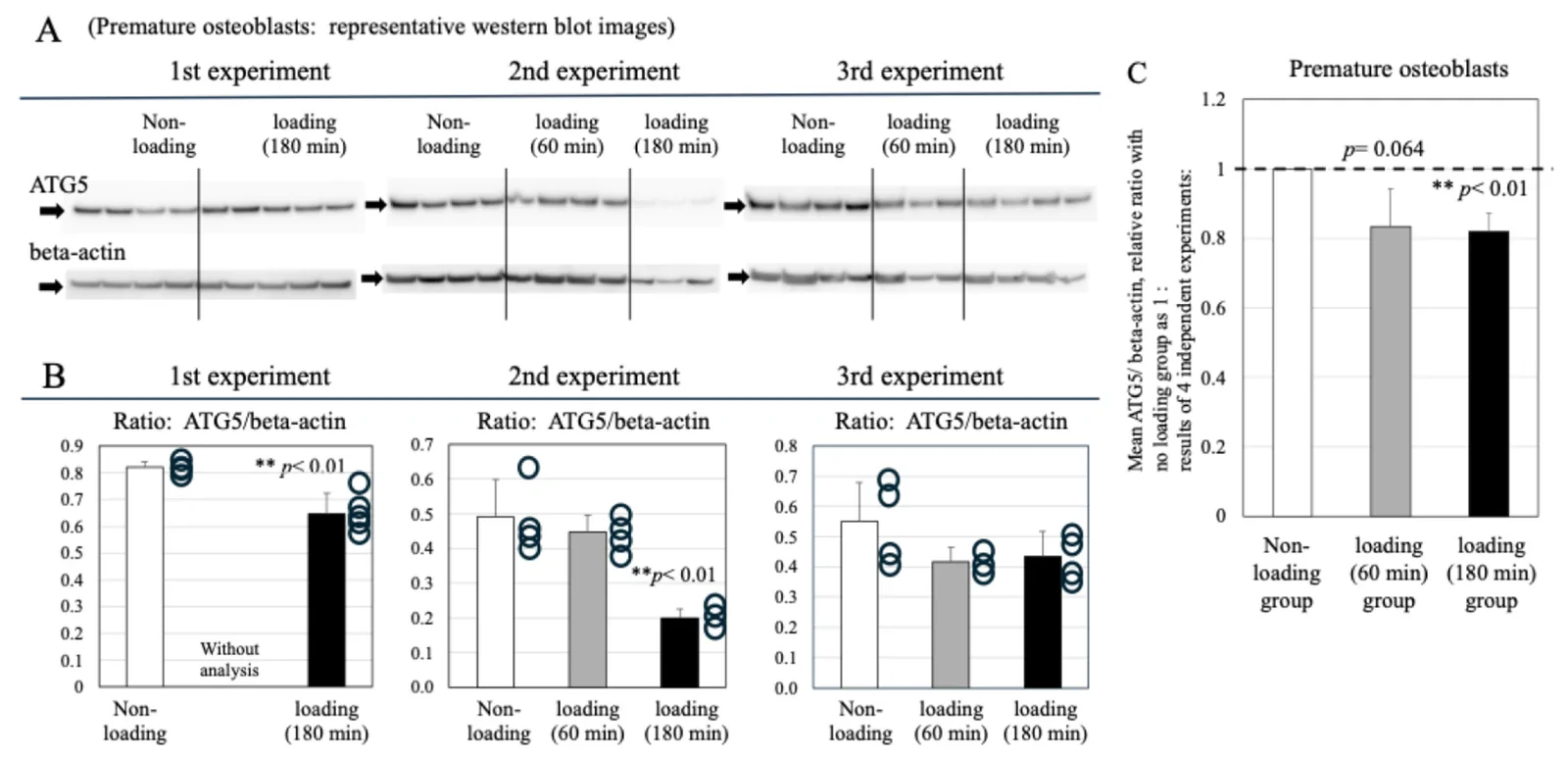

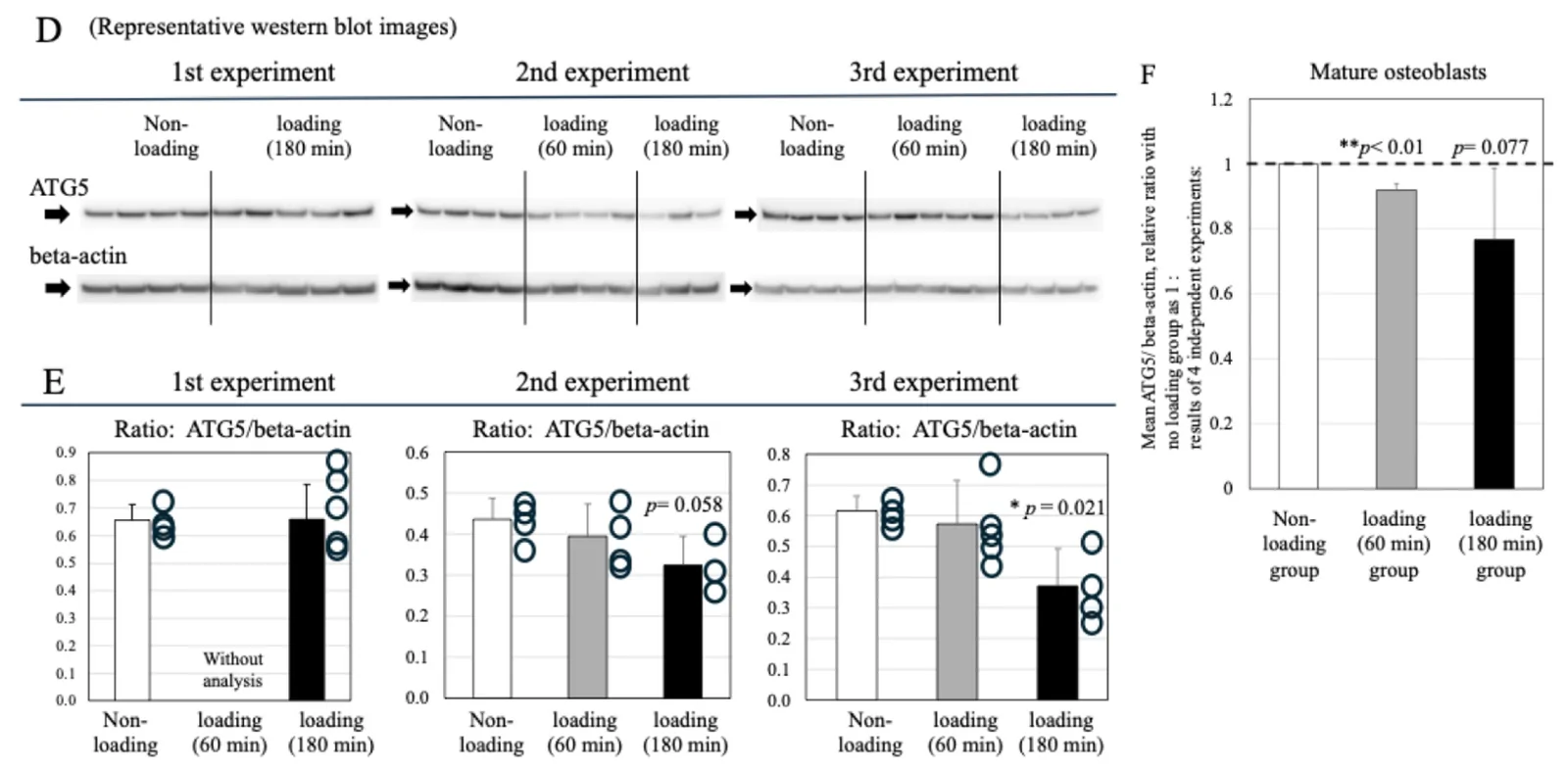
Modulation of ATG5 protein expression in premature human osteoblasts by cyclic strain. (**A**): Representative immunoblot images of ATG5 expression in static control, 60-min loaded, and 180-min loaded premature osteoblasts across three independent biological replicates (*n* = 3 distinct cell passages). (**B**): Relative densitometric ratios normalized to β-actin. ATG5 levels showed a decreasing trend in 180-min loaded premature osteoblasts compared with controls in the 1st (** *p* < 0.01) and 2nd (** *p* < 0.01) experimental runs. Individual scatter points represent separate data points. (**C**): Mean relative ATG5/β-actin expression ratios (control set as 1.0) derived from three independent biological replicates (*n* = 3). Values represent mean ± standard deviation. Cyclic compression for 60 or 180 min suppressed ATG5 levels in premature osteoblasts (control vs. 60-min load: *p* = 0.064; control vs. 180-min load: ** *p* < 0.01). Modulation of ATG5 protein expression in mature human osteoblasts by cyclic strain. (**D**): Representative Western blot images showing ATG5 band intensity in static control, 60-min loaded, and 180-min loaded mature osteoblasts (*n* = 3 independent biological runs). (**E**): Relative densitometric ratios normalized to β-actin. ATG5 levels demonstrated a declining trend in 180-min loaded mature osteoblasts compared with controls in the 2nd (*p* = 0.058) and 3rd (* *p* = 0.021) experimental runs. Individual scatter points denote separate data points. (**F**): Mean relative ATG5/β-actin ratios (control baseline = 1.0) across three biological replicates (*n* = 3). Data represent mean ± standard deviation. Dynamic strain for 60 or 180 min reduced ATG5 expression in mature osteoblasts compared with static controls (control vs. 60-min load: ** *p* < 0.01; control vs. 180-min load: *p* = 0.077).

As shown in Figure 5A,B, ATG5 protein expression in premature osteoblasts exhibited a downward trend under cyclic mechanical strain. Exposure to 180 min of compression significantly downregulated ATG5 signal intensity compared with static controls (*n* = 3 independent biological runs; control vs. 180-min load: *p* < 0.01; Figure 5C). Although 60-min strain did not reach statistical significance, a clear trend toward reduced ATG5 accumulation was evident (control vs. 60-min load: *p* = 0.064; Figure 5C).

As shown in Figure 5D,E, repetitive compressive loading also induced a decrease in ATG5 expression in mature osteoblasts. Repetitive compressive loading for 60 min significantly downregulated expression of ATG5 in mature osteoblasts compared to non-loaded controls (control vs. 60-min loading: *p* < 0.01, Figure 5F) while treatment for 180 min also showed a tendency to reduce ATG5 expression in mature osteoblasts (control vs. 180-min loading: *p* = 0.077, Figure 5F).

### 2.6. Effect of Physiologic and Repetitive Compressive Loading on Levels of the Autophagy-Related Protein Beclin-1 in Premature and Mature Osteoblasts

**Figure 6:**
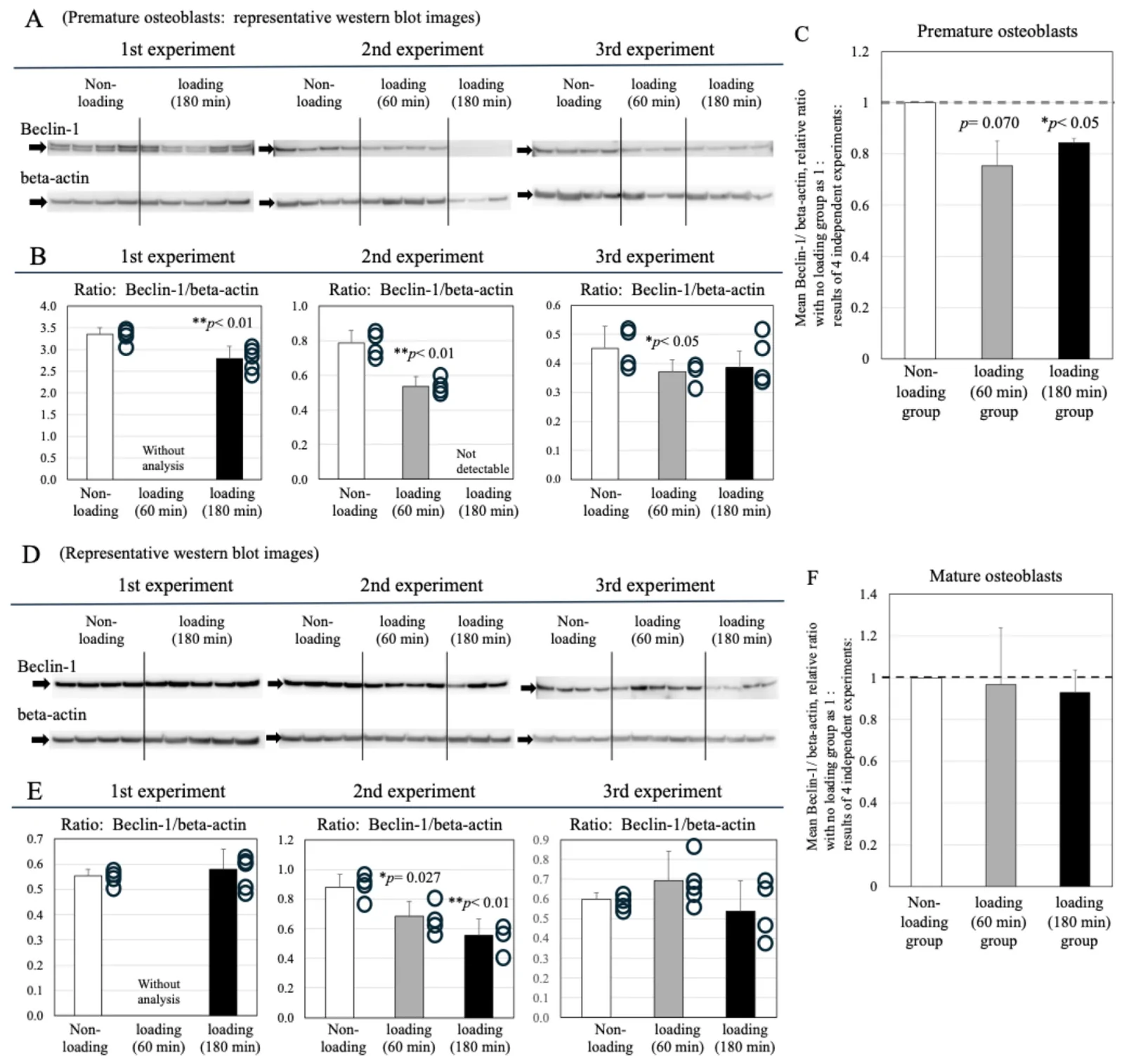
Effects of physiologic and repetitive compressive loading on expression of Beclin-1 in premature osteoblasts. (**A**): Immunoblot detection of Beclin-1 in static control, 60-min loaded, and 180-min loaded premature osteoblasts across three independent biological passages (*n* = 3). (**B**): Densitometric analysis normalized to β-actin. Beclin-1 levels showed a decreasing trend post-loading compared with controls [1st run (180-min load: ** *p* < 0.01), 2nd run (60-min load: ** *p* < 0.01; 180-min load: below detection limit), 3rd run (60-min load: * *p* < 0.05)]. Scatter points represent individual data points. (**C**): Mean relative Beclin-1/β-actin expression ratios (control= 1.0) across three biological replicates (*n* = 3). Data express mean ± standard deviation. Dynamic loading decreased Beclin-1 expression in premature osteoblasts (control vs. 60-min load: *p* = 0.070; control vs. 180-min load: * *p* < 0.05). Effect of cyclic compressive strain on Beclin-1 expression in mature osteoblasts. (**D**): Immunoblot images of Beclin-1 signal intensity in static control, 60-min loaded, and 180-min loaded mature osteoblasts (*n* = 3 independent biological replicates). (**E**): Densitometric analysis normalized to β-actin. Beclin-1 accumulation trended downward in loaded mature osteoblasts in the 2nd run (control vs. 60-min load: * *p* = 0.027; control vs. 180-min load: ** *p* < 0.01). Scatter points show individual values. (**F**): Mean relative Beclin-1/β-actin expression ratios (control baseline = 1.0) derived from three biological replicates (*n* = 3). Data express mean ± standard deviation. Cyclic compression for 60 or 180 min caused no statistically significant decrease in overall Beclin-1 levels in mature osteoblasts compared with non-loaded controls.

In premature osteoblasts, cyclic compressive strain induced a decrease in Beclin-1 protein expression compared with static baselines across experimental runs (Figure 6A,B). Following 60 min of loading, Beclin-1 levels trended downward (control vs. 60-min load: *p* = 0.070; Figure 6C), whereas 180 min of dynamic compression significantly suppressed Beclin-1 expression relative to non-loaded controls (control vs. 180-min load: * *p* < 0.05; Figure 6C).

In contrast to premature osteoblasts, in mature osteoblasts, no significant differences in Beclin-1 expression levels were observed between the loading and non-loading groups in either the 60-min or the 180-min loading experiments (Figure 6D–F).

### 2.7. Impact of Cyclic Mechanical Strain on Parkin Mitophagic Marker Levels

**Figure 7:**
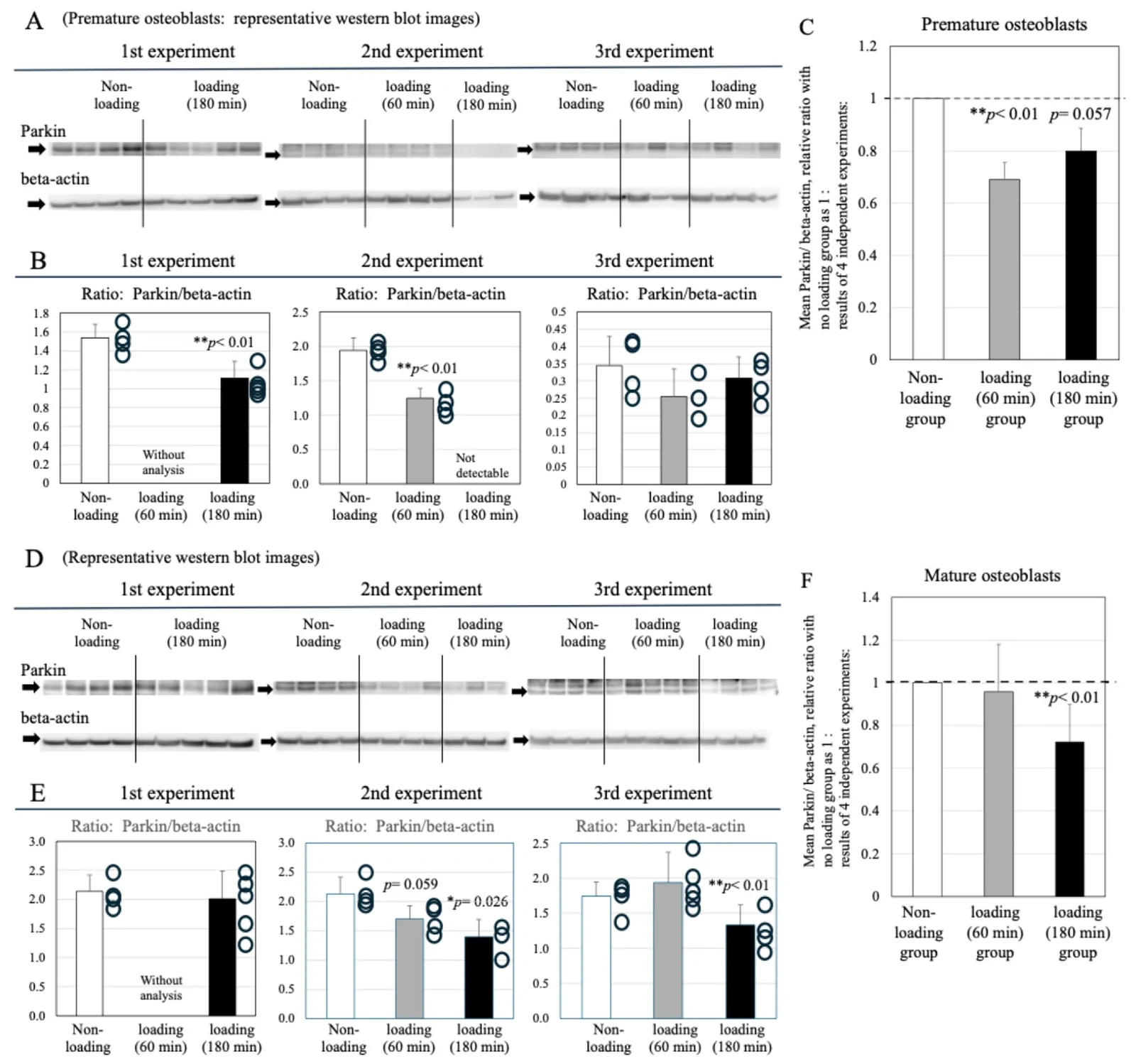
Influence of cyclic compressive strain on Parkin expression in premature osteoblasts. (**A**): Representative Western blot images showing Parkin signal intensity in static control, 60-min loaded, and 180-min loaded premature osteoblasts (*n* = 3 independent cell passages). (**B**): Densitometric evaluation normalized to β-actin. Parkin expression dropped in loaded premature osteoblasts compared with controls in the 1st run (180-min load: ** *p* < 0.01) and 2nd run (60-min load: ** *p* < 0.01; 180-min load: below detection threshold). Individual scatter points represent separate data points. (**C**): Mean relative Parkin/β-actin ratios (control baseline = 1.0) across three independent biological replicates (*n* = 3). Values represent mean ± standard deviation. Cyclic loading suppressed Parkin expression in premature osteoblasts (control vs. 60-min load: ** *p* < 0.01; control vs. 180-min load: *p* = 0.057). Influence of cyclic compressive strain on Parkin expression in mature osteoblasts. (**D**): Immunoblot detection of Parkin in static control, 60-min loaded, and 180-min loaded mature osteoblasts (*n* = 3 independent biological runs). (**E**): Relative densitometric ratios normalized to β-actin. Parkin expression showed a decreasing trend post-loading in the 2nd run (control vs. 60-min load: *p* = 0.059; control vs. 180-min load: * *p* = 0.026) and 3rd run (control vs. 180-min load: ** *p* < 0.01). Scatter points show individual values. (**F**): Mean relative Parkin/β-actin ratios (control baseline = 1.0) across three biological replicates (*n* = 3). Data denote mean ± standard deviation. Cyclic strain for 180 min significantly decreased Parkin expression in mature osteoblasts compared with static controls (** *p* < 0.01).

In premature osteoblasts, dynamic compressive loading resulted in marked downregulation of Parkin expression across individual experimental runs in both the 60-min and 180-min loading cohorts relative to static controls (Figure 7A,B) (*n* = 3 independent biological replicates; control vs. 60-min load: ** *p* < 0.01; control vs. 180-min load: *p* = 0.057; Figure 7C).

In mature osteoblasts, repetitive compression similarly reduced Parkin protein accumulation (Figure 7D,E). Exposure to 60 min of loading produced a trend toward decreased Parkin levels without reaching statistical significance. Conversely,180 min of continuous dynamic compression significantly downregulated Parkin expression in mature osteoblasts compared with unloaded controls (control vs. 180-min load: ** *p* < 0.01; Figure 7F).

## 3. Discussion

Elucidating the cellular mechanisms by which bone and cartilage adapt to mechanical strain is fundamental to discovering novel therapeutic targets for age-related musculoskeletal disorders, including osteoporosis and osteoarthritis (OA). It is established that osteocytes entombed within the mineralized bone matrix act as the principal mechanosensors driving mechanical load-induced bone formation [9,10,14,15,16,17,18,19]. Sclerostin, an osteocyte-derived protein, serves as a pivotal molecular switch in this mechanotransductive cascade [15,16,17,18]. Under physiological loading, sclerostin production is suppressed, unleashing canonical Wnt/ββ-catenin signaling in neighboring osteocytes and surface osteoblasts to stimulate osteogenic differentiation and bone synthesis [15,19,20]. This intercellular crosstalk via the sclerostin–Wnt/ββ-catenin axis is considered indispensable for force-induced bone anabolic responses.

In contrast to this classical osteocyte-mediated signaling paradigm, our previous investigations demonstrated that mechanical stress can directly trigger osteoblast differentiation and bone matrix deposition independently of sclerostin–Wnt/ββ-catenin intercellular pathways [22,23]. Specifically, mechanical strain activates membrane-bound glucose transporter 1 (Glut1) on osteoblasts to enhance glucose uptake, which in turn modulates the intracellular energy sensor sirtuin 1 (SIRT1) and derepresses the key osteogenic transcription factor Runx2 [24]. This Glut1–SIRT1–Runx2 metabolic axis establishes that osteoblasts possess intrinsic mechanosensory capability, directly reconfiguring cellular energy metabolism and osteogenic activity [24]. Because osteoblasts ultimately execute bone formation whether stimulated directly or via osteocytic signals, clarifying their autonomous stress-adaptation capacity—particularly their internal protective defense mechanisms—is of critical pathophysiological importance.

We therefore investigated the stress-defense capacity of subchondral bone osteoblasts, which, like articular chondrocytes, are continuously exposed to cyclic mechanical loads. The contribution of subchondral bone remodeling to osteochondral joint damage in OA has received increasing clinical and basic research attention [37,38,39]. Pathological alteration of the subchondral architecture is recognized as a hallmark feature of OA development [37,40]. Composed of the subchondral plate and underlying trabecular bone, this tissue is structurally, biomechanically, and biochemically linked to the overlying articular cartilage [37]. Mechanical overload disrupts subchondral bone turnover prior to overt cartilage degradation, with subchondral osteoblasts and osteocytes acting as primary mechanosensors [38,39]. Under pathological strain, subchondral osteoblasts facilitate disease progression across the bone–cartilage interface, making osteochondral crosstalk a crucial factor in OA pathogenesis.

Consequently, understanding how subchondral osteoblasts protect themselves against mechanical stress is vital. Autophagy serves as a selective degradation mechanism that clears damaged proteins and dysfunctional organelles, maintaining metabolic quality control and cellular homeostasis. The present study demonstrates that repetitive mechanical loading downregulates the expression of key autophagic regulatory factors—ATG5, Beclin-1, and Parkin—in subchondral osteoblasts. In our 3D culture system, dynamic compressive loading for 60 or 180 min significantly suppressed these three regulators compared with unloaded conditions. Given that ATG5 and Beclin-1 are fundamental to autophagosome initiation and maturation [44,45,46,47], mechanical stress-induced suppression of these factors likely impairs the clearance of damaged organelles, resulting in the intracellular accumulation of cytotoxic protein aggregates in subchondral osteoblasts. Mechanistically, ATG5 forms a ubiquitin-like complex with ATG12 to drive autophagosomal membrane elongation [44], while also participating in apoptotic signaling via cytoskeletal interactions [45]. Beclin-1 coordinates with Vps34 and Vps15 to form a class III phosphatidylinositol 3-kinase complex that initiates autophagic vesicle formation [46,47]. Our findings demonstrate that physiological levels of repetitive compressive strain suppress both ATG5 and Beclin-1 in subchondral osteoblasts, impairing basic autophagic capacity and threatening cell survival within the subchondral bone microenvironment.

In addition to core autophagic machinery, repetitive compressive loading reduced the expression of Parkin, a central regulator of mitophagy. This aligns with our previous observation in OA chondrocytes, where mechanical stress suppressed Parkin-mediated mitophagy [36]. Parkin selectively targets depolarized, damaged mitochondria for autophagic clearance, maintaining mitochondrial quality control across diverse tissues [48,49]. Because mitochondrial dysfunction is closely linked to cellular senescence and degenerative joint diseases like OA [50], Parkin depletion in subchondral osteoblasts under mechanical strain likely impairs mitochondrial turnover, disrupting cellular energetic homeostasis in subchondral bone.

Ultrastructural TEM analysis confirmed this autophagic impairment. Unloaded controls (both premature and mature osteoblasts) exhibited abundant autophagosomes, lysosomes, autophagosome–lysosome fusion events, and mature autolysosomes, reflecting active baseline organelle turnover. Conversely, repetitive compressive loading caused a marked reduction in autophagosomes and autolysosomes in both osteoblast maturation stages. Notably, premature osteoblasts subjected to 180 min of continuous compressive loading lacked distinct autophagic structures and instead displayed widespread accumulation of undegraded, damaged organelles. Because autophagosomal-lysosomal degradation normally recycles cellular debris into basic amino acids and metabolites for structural maintenance, these TEM observations indicate that physiological levels of repetitive strain suppress the autophagic degradation machinery in subchondral osteoblasts.

To fully delineate these autophagic dynamics, it is important to distinguish whether autophagosome alterations reflect accelerated autophagic induction or blocked autolysosomal clearance. Measuring autophagic flux will be essential to clarify the precise stage at which mechanical stress disrupts vesicle trafficking. Future studies should combine confocal fluorescence microscopy with autophagosome/lysosome-specific probes and lysosomal acidification inhibitors. Overcoming the technical challenge of performing live-cell confocal imaging within 3D collagen sponge matrices will be a primary technical objective.

Furthermore, because Parkin-mediated mitophagy involves multi-step molecular machinery, definitive proof of mitophagy impairment requires comprehensive functional assays, such as mitochondrial membrane potential tracking, mitochondrial–LC3 colocalization, and mitophagy reporter constructs. However, conducting these measurements via flow cytometry, microplate readers, or standard fluorescence microscopy is technically constrained by the difficulty of isolating viable, morphologically intact osteoblasts from 3D collagen scaffolds post-loading. Developing in situ analytical methods capable of evaluating dynamic organelle turnover directly within intact 3D constructs remains an ongoing priority.

Similarly, evaluating mitochondrial functional status under mechanical strain requires direct assessments of mitochondrial membrane potential (hyperpolarization/depolarization), reactive oxygen species (ROS/mito-ROS) generation, and mitochondrial DNA integrity. Because real-time confocal imaging of live osteoblasts embedded in 3D collagen gels remains methodologically limited, we evaluated indirect energetic markers using total cellular protein extracts, as presented in Supplementary Data. These analyses revealed that repetitive compressive loading significantly reduced cellular ATP content and mitochondrial NAD activity in subchondral osteoblasts. While these biochemical assays confirm energetic impairment, we acknowledge the need to establish advanced imaging methodologies to directly observe dynamic mitochondrial flux under strain.

Interestingly, clinical applications often employ biophysical energy—such as piezoelectric and triboelectric stimulation—to enhance intrinsic bone regeneration [12]. Mechanically induced potential differences in bone tissue stimulate osteocytes, osteoblasts, and progenitor cells, promoting an osteogenic microenvironment [12,13]. Consistent with this anabolic potential, our study showed that repetitive compressive strain increased alkaline phosphatase (ALP) production in both premature and mature osteoblasts, indicating that mechanical force promotes osteogenic differentiation and functional activity. Furthermore, TEM imaging of mature osteoblasts subjected to 180 min of loading revealed dense intracellular collagen fibril assembly, indicating active osteogenic matrix synthesis. These observations align with clinical evidence supporting mechanical stimulation for bone repair.

Our cumulative findings present an intriguing physiological scenario: repetitive mechanical compression stimulates osteoblastic functional activity (increased ALP and matrix production) while simultaneously suppressing autophagic and mitophagic quality control (decreased ATG5, Beclin-1, and Parkin). We propose two potential, non-mutually exclusive explanations for this temporal and functional discrepancy between osteogenic differentiation and autophagic clearance:

1. Stage-dependent physiological reprogramming: The downregulation of autophagy factors may reflect a natural shift in cellular program during osteogenic maturation rather than acute pathological breakdown. In support of this concept, RUNX2 overexpression in MC3T3-E1 pre-osteoblasts was reported to elevate ALP, osteocalcin, and mineralization while concurrently suppressing Beclin-1 and ATG5 expression [51]. Although that study did not apply mechanical strain, it demonstrates that osteogenic differentiation and autophagic suppression can be biologically co-regulated during bone matrix synthesis.
2. Exhaustion of homeostatic capacity: Prolonged mechanical strain may push osteoblasts beyond their adaptive threshold. While osteoblasts initially mount an anabolic response (upregulating ALP) to adapt to physical strain, sustained loading may overwhelm the autophagic recycling network, leading to a progressive decline in organelle quality control. Over time, this unresolved organelle damage could impair long-term osteoblast viability.

In summary, repetitive mechanical strain applied to subchondral bone osteoblasts promotes osteogenic activation while compromising autophagic defense mechanisms. While mechanical loading generally exerts an anabolic, bone-forming effect across skeletal tissue, subchondral bone near joint interfaces experiences intense, localized cyclic loading. Under these conditions, the strain-induced decline in autophagic organelle clearance causes defective mitochondria and damaged proteins to accumulate in subchondral osteoblasts. This unresolved cellular stress may drive subchondral bone degeneration, ultimately contributing to the onset and progression of OA.

## 4. Materials and Methods

### 4.1. Primary Human Subchondral Osteoblast Culture and Differentiation

Commercial human subchondral osteoblasts derived from the hip joint of a 64-year-old Caucasian female donor were acquired from PromoCell GmbH (Heidelberg, Germany). These cells were supplied as cryopreserved cell pellets comprising undifferentiated, mesenchymal progenitor-like osteoblasts. In accordance with manufacturer specifications, osteoblastic differentiation and maturation were induced using specialized media. Cryopreserved stocks stored at −80 °C were thawed and differentiated separately across three independent biological replicate runs (three distinct cell batches).

Briefly, immature cells were initially expanded in Osteoblast Growth Medium (PromoCell GmbH) for 7–9 days. To induce functional mineralization and complete osteogenic maturation, the culture medium was transitioned to Osteoblast Mineralization Medium (PromoCell GmbH) from day 7–9 up to day 22–23. Environmental conditions were maintained at 37 °C with 5% CO_2_ in a humidified incubator. Ethical clearance for the study protocol was granted by the Ethics Committee of St. Marianna University School of Medicine (Approval No. 1315). All procedures strictly adhered to institutional ethical guidelines and the principles of the Declaration of Helsinki (1975, revised 2000).

### 4.2. Fabrication of 3D Osteoblast–Collagen Sponge Constructs

To establish three-dimensional (3D) tissue constructs, osteoblasts at distinct differentiation stages (pre-osteoblasts collected on days 7–9; mature osteoblasts collected on days 22–23) were harvested from monolayer cultures and suspended in Osteoblast Growth Medium, modifying previously described protocols [24,52]. Porous collagen sponges (AteloCell^®^; Koken Co., Ltd., Tokyo, Japan; dimensions: 5 mm diameter × 3 mm height; mean pore size: ~100 µm, range: 50–200 µm) were positioned within 96-well culture plates (Iwaki AGC Techno Glass Co., Ltd., Shizuoka, Japan). Each scaffold was inoculated with 1 × 10^5^ cells in suspension. Constructs were maintained under standard incubation conditions (37 °C, humidified air with 5% CO_2_). Following scaffold seeding, the resulting 3D cell constructs were equilibrated in osteoblast growth medium for 12 h prior to experimental loading.

### 4.3. Application of Repetitive Compressive Stress

The prepared 3D constructs were transferred to 24-well plates (Iwaki AGC Techno Glass) filled with 1.5 mL of Osteoblast Mineralization Medium (PromoCell GmbH) per well. Dynamic compressive loading was delivered using a custom-engineered loading system (Figure 1), following the loading methodology described by Sato et al. [36]. Torque tracking was governed by a precision servo-motor system operating within a ±1% error tolerance. Continuous 72-h validation tests confirmed system stability, yielding a cycle-to-cycle coefficient of variation (CV) of 0.9%.

Physiological cyclic compression was delivered at either 0 kPa (static control) or 2.5 kPa (25.5 gf/cm^2^) [24,36,51,52]. Loading protocols were executed for duration periods of 60 or 180 min at a frequency of 52 cycles/minute within a humidified incubator (37 °C, 5% CO_2_). Non-loaded constructs served as controls. Immediately following stress application, conditioned culture supernatants and cellular samples were harvested and preserved at −80 °C for downstream biochemical analyses.

### 4.4. Extraction and Quantification of Intracellular Protein

Intracellular total protein was harvested from 3D constructs immediately post-loading according to established procedures [24,52]. Samples were thoroughly rinsed thrice using ice-cold phosphate-buffered saline (PBS) and homogenized in T-PER™ Tissue Protein Extraction Reagent (Thermo Fisher Scientific, Waltham, MA, USA) supplemented with cOmplete™ protease inhibitor cocktail (Merck–Millipore, Darmstadt, Germany). To release protein fractions, the tissue mixture underwent ultrasonic disruption followed by centrifugation at 25,000× *g* for 5 min at 4 °C. Supernatants containing extracted cellular proteins were collected for functional assays.

### 4.5. Immunoblotting Analysis

Intracellular expression of autophagy-associated markers (Parkin, ATG5, and Beclin-1) in response to repetitive loading was evaluated via Western blot, adapting methodologies from previous studies [24,52]. Assays were conducted using cell lysates derived from three independent biological replicates (*n* = 3 distinct cell passages) per experimental condition.

Protein samples (6 μg/lane) were resolved by 10% sodium dodecyl sulfate–polyacrylamide gel electrophoresis (SDS-PAGE) and electrotransferred onto membranes. Target proteins were probed using primary antibodies: mouse anti-human Parkin monoclonal (1:1000; Santa Cruz Biotechnology, Santa Cruz, CA, USA), rabbit anti-human ATG5 polyclonal (1:1000; Cell Signaling Technology, Danvers, MA, USA), and rabbit anti-human Beclin-1 polyclonal (1:1000; Cell Signaling Technology). Horseradish peroxidase (HRP)-conjugated secondary antibodies were applied accordingly: HRP-anti-mouse IgG for Parkin (1:5000; DAKO A/S, Glostrup, Denmark) and HRP-anti-rabbit IgG for ATG5 and Beclin-1 (1:4000; DAKO A/S). An HRP-conjugated mouse anti-ββ-actin monoclonal antibody (1:10,000; Proteintech Group, Rosemont, IL, USA) was employed as an internal loading reference. Chemiluminescent signals were captured, and band intensities quantified using an extended cavity laser imaging system (GE Healthcare Bio-Sciences KK, Tokyo, Japan).

### 4.6. Assessment of Osteoblast Functional Marker Activity

To assess cell-type-specific metabolic responses to mechanical strain, osteogenic functional activity was quantified following cyclic loading of 3D sponge constructs. Cell lysates and conditioned media were harvested post-treatment.

Alkaline phosphatase (ALP) expression levels, serving as a functional indicator of osteoblastic phenotype, were determined using a colorimetric assay kit (Alkaline Phosphatase Assay Kit; Abcam Limited, Cambridge, UK). Absorbance values were measured at 450 nm using a microplate spectrophotometer (Multiskan™ FC Microplate Photometer, Thermo Fisher Scientific).

### 4.7. Ultrastructural Observation via Transmission Electron Microscopy (TEM)

Ultrastructural changes and autophagic structures within 3D cell constructs subjected to or spared from mechanical compression were evaluated using TEM. Samples underwent sequential fixation with 2% glutaraldehyde, 1%osmium tetroxide, and 1% aqueous tannic acid solution. Microscopic imaging was conducted using a Hitachi S-4800 transmission electron microscope (Hitachi Ltd., Tokyo, Japan) following the loading methodology described by Sato et al. [36].

To rigorously prevent pseudoreplication and guarantee statistical independence, biological replicates (three independent cell/sponge batches per group; N = 3) were defined as the statistical unit. Three to four cells were randomly selected and imaged per construct, yielding 10 evaluated cells per group. Autophagosomes, lysosomes, and autolysosomes were enumerated independently by three researchers blinded to experimental conditions. The average count across observers was compiled for each cell. Construct-level mean values were subsequently derived from these data, and statistical differences between loaded and control conditions were evaluated using a two-tailed Student’s *t*-test.

## 5. Conclusions

Our data demonstrate that reductions in both mitophagy and cellular autophagy in response to repetitive mechanical stress, even at a physiologic level, may lead to accumulation of defective mitochondria and abnormal cellular proteins in the cells, thus reducing osteoblast activity and affecting the homeostasis of subchondral bone tissue, and ultimately contributing to OA progression.

## Data Availability

The original contributions presented in this study are included in the article/Supplementary Materials. Further inquiries can be directed to the corresponding author.

## Supplementary Materials

The following supporting information can be downloaded at https://www.mdpi.com/article/10.3390/ijms27219903/s1.
