# Supplementary material for: Effects of Repetitive Compressive Loading on the Expression of Autophagy-Related Factors, Autophagy Capacity and Cellular Activity in Human Subchondral Bone Osteoblasts
Source: Int J Mol Sci. 2026 Nov 5;27(21):9903. doi: 10.3390/ijms27219903 (PMC13764388; doi:10.3390/ijms27219903)
Supplement: Supplementary file 1 [file ijms-27-09903-s001.zip › ijms-4556884-supplementary.pdf]

Supplementary data

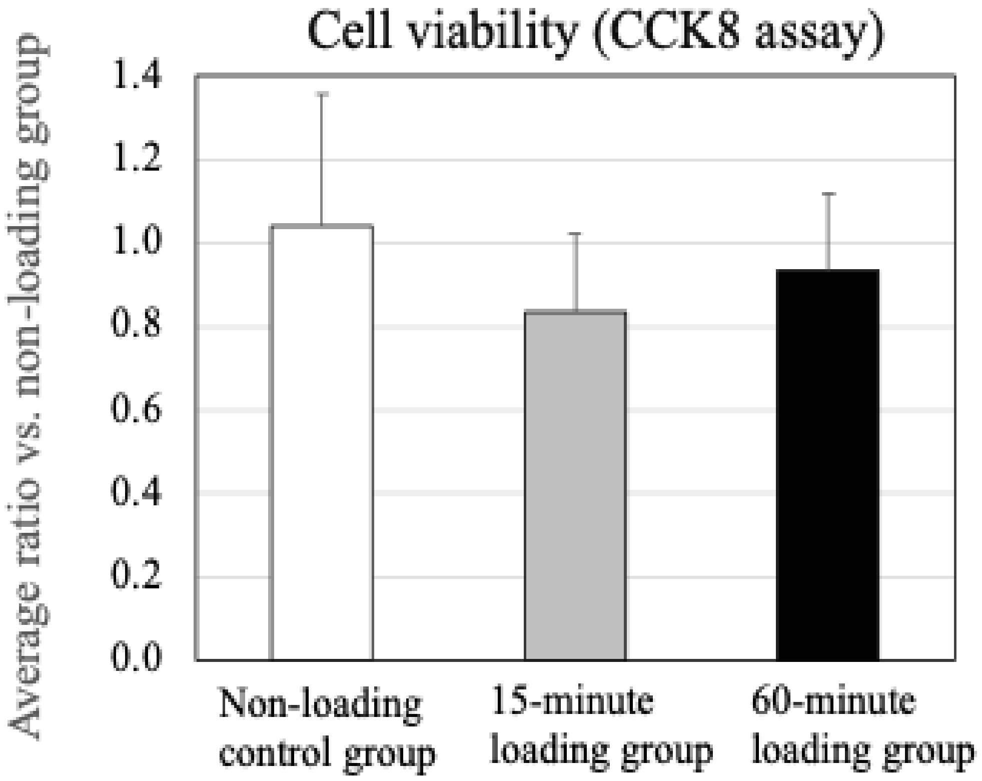

Figure S1. Effects of physiologic and repetitive compressive loading on cell viability in the 3D cell-culture construct after mechanical loading.

Cell viability was analyzed using the CCK-8 assay. A load of 2.5 kPa did not adversely affect cell viability.

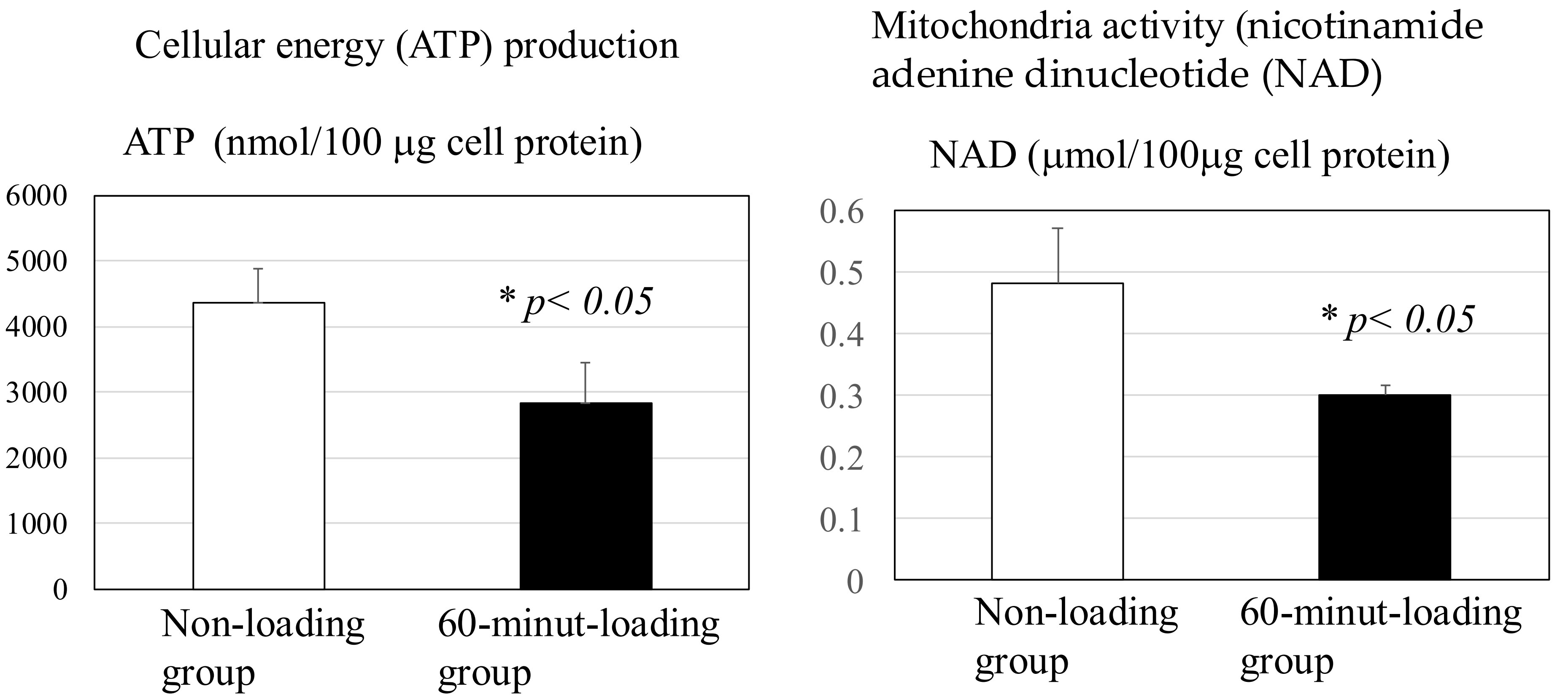

Figure S2. Effects of physiological and repetitive compressive loading on ATP production and nicotinamide adenine dinucleotide (NAD) activity in osteoblasts.

Significant differences in ATP production and NAD activity in osteoblasts were observed between the unloaded control group and the group subjected to 60 minutes of loading.
